# Absence of IFNγ expression induces neuronal degeneration in the spinal cord of adult mice

**DOI:** 10.1186/1742-2094-7-77

**Published:** 2010-11-12

**Authors:** Sheila CS Victório, Leif A Havton, Alexandre LR Oliveira

**Affiliations:** 1 Department of Anatomy, Cell Biology, Physiology and Biophysics, Institute of Biology, University of Campinas (UNICAMP), CP 6109, CEP 13083-970, Campinas, SP, Brazil; 2 Department of Neurology, University of California, Los Angeles

## Abstract

**Background:**

Interferon gamma (IFNγ) is a pro-inflammatory cytokine, which may be up-regulated after trauma to the peripheral or central nervous system. Such changes include reactive gliosis and synaptic plasticity that are considered important responses to the proper regenerative response after injury. Also, IFNγ is involved in the upregulation of the major histocompatibility complex class I (MHC class I), which has recently been shown to play an important role in the synaptic plasticity process following axotomy. There is also evidence that IFNγ may interfere in the differentiation and survival of neuronal cells. However, little is known about the effects of IFNγ absence on spinal cord neurons after injury.

**Methods:**

We performed a unilateral sciatic nerve transection injury in C57BL/6J (wild type) and IFNγ-KO (mutant) mice and studied motoneuron morphology using light and electron microscopy. One week after the lesion, mice from both strains were sacrificed and had their lumbar spinal cords processed for histochemistry (n = 5 each group) and transmission electron microscopy (TEM, n = 5 each group). Spinal cord sections from non-lesioned animals were also used to investigate neuronal survival and the presence of apoptosis with TUNEL and immunohistochemistry.

**Results:**

We find that presumed motoneurons in the lower lumbar ventral horn exhibited a smaller soma size in the IFNγ-KO series, regardless of nerve lesion. In plastic embedded sections stained with toluidine blue, the IFNγ-KO mice demonstrated a greater proportion of degenerating neurons in the ventral horn when compared to the control series (p < 0.05). Apoptotic death is suggested based on TUNEL and caspase 3 immunostaining. A sciatic nerve axotomy did not further aggravate the neuronal loss. The cellular changes were supported by electron microscopy, which demonstrated ventral horn neurons exhibiting intracellular vacuoles as well as degenerating nuclei and cytoplasm in the IFNγ-KO mice. Adjacent glial cells showed features suggestive of phagocytosis. Additional ultrastructural studies showed a decreased number of pre-synaptic terminals apposing to motoneurons in mutant mice. Nevertheless, no statistical difference regarding the input covering could be detected among the studied strains.

**Conclusion:**

Altogether, these results suggest that IFNγ may be neuroprotective and its absence results in neuronal death, which is not further increased by peripheral axotomy.

## Background

Transection of a peripheral nerve results in a complex retrograde reaction in the spinal cord, involving motoneurons, glia and immune cells. Lesioned nerve cells signal to pre-synaptic terminals leading to an intense rearrangement of synapses. The precise mechanisms behind such synaptic plasticity are not clearly understood, although certain molecules certainly influence the process. One of these molecules is the major histocompatibility complex of class I (MHC I), that is vigorously upregulated in spinal motoneurons in the acute phase following peripheral axotomy. Importantly, interferon gamma (IFNγ), a pro-inflammatory cytokine, is the most potent inducer of MHC I expression and is upregulated in the CNS after injury [1,2]. It is also present at elevated levels during the course of autoimmune diseases, such as the multiple sclerosis, and in chronic neurodegenerative diseases [3,4].

CD4+, CD8+ T and natural killer (NK) cells are the major source of IFNγ [5], but there is evidence [5] that this cytokine is produced within the nervous system by neurons and glial cells, in the absence of infiltrating immune cells. Taking into account that the IFNγ receptor is detected in neurons and glial cells [2,6], auto/paracrine roles may be of relevance to the response to injury.

The rearrangement of synaptic inputs to motoneurons following peripheral axotomy has been investigated for several years. It has been proposed that the initial loss of synapses, observed within the first week post lesion, may represent a neuronal strategy to survive the transection of the axon as well as to avoid excitotoxicity by glutamate. Also, it is possible that such partial disconnection from the spinal network may facilitate the regenerative process, since cytoskeleton proteins, such as protein-43 (GAP43) and neurofilaments, are actively synthesized. In this regard, we have demonstrated that the increase of synaptic retraction in spinal motoneurons by exogenous treatment with interferon beta correlates to a faster and more effective axonal regeneration in C57BL/6J mice [7]. The enhancement of synaptic plasticity after IFN beta treatment was linked to an increased astroglial reaction, as seen both *in vivo *and *in vitro *and with an upregulation of MHC I by motoneurons and astrocytes [8]. It is possible, in this way, that the absence or interference in the level of certain molecules, such as cytokines and neurotrophic factors, may directly alter the neuronal response to injury and ultimately influence the survival and regeneration process [9].

Although pro-inflammatory cytokines function as a mediator in the pathogenesis of the inflammatory lesion or work cooperatively to promote neuronal death [10], some evidence for beneficial properties of IFNγ has recently emerged. Temporal expression of IFNγ may suggest an important function during the development [6] and also to be needed during regenerative events after trauma [11]. IFNγ has been shown to control phosphorylation and the nuclear translocation of STAT I, to regulate MHC class I gene expression [6,12], and to influence neuronal excitability by inducing the expression of the peripheral nerve-type sodium channel gene PN1 [13]. Moreover, the absence of IFNγ in deficient mice induced the death of hippocampal CA1 neurons after virus infection, suggesting a neuroprotective role of IFNγ in the brain [14].

Nevertheless, little is known about the effects of the absence of IFNγ on spinal cord neurons and spinal circuits in neurologically intact subjects and after nerve injury. Here, we studied the effects of a sciatic nerve transection injury on spinal motoneuron morphology in IFNγ-KO mice using light and transmission electron microscopy.

We show herein that presumed motoneurons in the lower lumbar ventral horn exhibited a smaller soma size in the IFNγ series, regardless of nerve lesion. Also, the IFNγ mice demonstrated a greater proportion of degenerating neurons in the ventral horn when compared to the control series.

## Methods

### Animals

Adult male C57BL/6J (wild type - wt) and C57BL/6J IFNγ-KO (mutant) mice (n = 15 each strain), 6-8 weeks old, were obtained from the Multidisciplinary Center of Biological Investigation (CEMIB/Unicamp) and housed under a 12-h light/dark cycle with free access to food and water. The Institutional Committee for Ethics in Animal Experimentation approved the study (CEEA/IB/Unicamp, proc. 1172-1), and the experiments were carried out in accordance with the guidelines of Brazilian College for Animal Experimentation (COBEA). In the present study, mice from both strains (n = 10 each) were subjected to unilateral sciatic nerve transection and kept alive for one week. The distribution of motoneurons in lamina IX was analyzed based on the frequency distribution of soma size. Also, we investigated a series of morphological changes present in motoneurons from IFNγ-KO mice. In all cases, the contralateral side of the spinal cord was used as control. In order to assess if the absence of IFNγ affects the surviving motoneurons, a group of unlesioned animals was also used (n = 5 for each strain).

### Surgical procedures and tissue preparation

The mice were anesthetized with a mixture of Kensol (xylasin, Köning, 10 mg/kg) and Vetaset (Cetamin, Fort Dogde, 50 mg/kg, 1:1, 0.12 mL/25 g, i.p.) and were subjected to a left sciatic nerve transection at the level of the obturator tendon. A 2 mm-long segment of the distal stump was removed to avoid regeneration. The muscular and the skin planes were sutured, and the animals were kept in the animal housing facility for one week. All the animals were then sacrificed with an overdose of anesthetic and subjected to trans-cardiac perfusion with 0.1 M PBS (20 ml, pH 7.4) and fixed with 10% formaldehyde in PBS for Nissl staining, immunohistochemistry and TUNEL analysis (animals without surgery) or with Karnovsky solution (2.5% glutaraldehyde and 0.5% paraformaldehyde in phosphate buffer pH 7.4) for electron microscopy (animals with surgery). The lumbar portion of the spinal cord was removed and frozen or embedded in resin.

### Cell count procedures

The lumbar spinal cord from unlesioned mice was frozen in liquid nitrogen at -40°C for cryostat sectioning (12 μm). The sections were transferred to gelatine-coated slides, which were stained for 5 min in an aqueous 1% cresyl fast violet at 50°C. The tissue was dehydrated and mounted in enthelan. Neurons localized in the ventral horn (lamina IX) were counted in every fourth section (20 sections/animal in each group). Only cells with a visible nucleus and nucleolus were counted. Bilateral counts were made in sections from the lumbar intumescence in each animal. The absolute and relative number of ventral horn degenerating neurons per section in each group were used to calculate the mean number of surviving cells per experimental group. To correct for any variation in neuronal size between groups, the Abercrombie's formula [15] was used:

N=nt/(t+d)

Where *N *is the corrected number of counted neurons, *n *is the counted number of cells, *t *is the thickness of the section (12 μm) and *d *is the average diameter of the cells. Since neuronal size significantly affects cell counts, the value of *d *was calculated specifically for each experimental group. For this purpose, the diameter of 15 randomly picked neurons from each group was measured using Image Tool software (Version 3.0, The University of Texas Health Center in Santo Antonio, TX, USA) and the mean value calculated.

### Immunohistochemistry

Spinal cord sections were obtained and incubated in TBS-T with 3% BSA at room temperature for 30 minutes. In sequence, the specimens were incubated overnight, at 4°C in a moist chamber, with a rabbit anti-cleaved caspase-3 antibody (1:400; Cell Signaling Technology, Danvers, MA, USA). After that, the slides were washed 3 times in Tris buffer plus 0.2% Tween (TBS-T), and blocked in TBS-T with 3% BSA at room temperature. The sections were then incubated with a CY3-conjugated secondary antibody (1:250) from Jackson Immunoresearch (Bar Harbor, ME, USA) for 1 h in a moist chamber at room temperature. The slides were then rinsed in TBS-T, mounted in a mix of glycerol/PBS (3:1), observed and documented under a fluorescence microscope (Eclipse TS100, Nikon, Tokio, Japan) equipped with a digital camera (DXM1200F, Nikon, Tokio, Japan).

### Staining of apoptotic cells (TUNEL)

Frozen spinal cord sections from unlesioned animals were post-fixed in ethanol/acetic acid (2:1) for 5 min at -20°C and rinsed twice for 5 min in PBS. The slides were transferred to a humidified chamber and the equilibration buffer solution was applied (Oncor, s7110-1) and incubated for 5 min at room temperature (RT). The equilibration buffer was shaken off and the TdT enzyme solution (Oncor, s7110-2 and 3) was applied for 60 min at 37°C. The reaction was stopped with the stop/wash solution (Oncor, s7110-4) for 30 min at 37°C. After washing in PBS for 10 min, the sections were incubated with the fluorescein solution (Oncor, s7110-5 and 6) for 30 min. The slides were rinsed in PBS and mounted with glass coverslips in a mix of glycerol/PBS (3:1) solution. Images were obtained using a fluorescence microscope (Eclipse TS100, Nikon, Tokio, Japan) equipped with a digital camera (DXM1200F, Nikon, Tokio, Japan).

### Morphometric analysis

The lumbar spinal cords were dissected out and stored overnight in fixative at 4^° ^C. The specimens were then osmicated, dehydrated and embedded in Durcupan (Fluka). Serial transverse sections (55-60 nm) from L4-L6 segments were obtained by using a PowerTome X ultramicrotome (RMC Products, Boeckeler Instruments, Tucson, AZ). Ultrathin sections were collected and stained with toluidine blue. For morphometric analysis, bilateral ventral horns of two sections from each lumbar spinal cord were photographed with Micropublisher 5 megapixel digital camera (Q Imaging, Burnaby, BC) attached to a Nikon E600 microscope and only the motoneurons present in lamina IX and cut at the nuclear plane were included in the analysis. The mean soma diameter was calculated with the C-Imaging software (Compix Inc., Sewickley, PA) as the average between the longest soma diameter and the longest perpendicular diameter. These measurements were used to assess the size distribution pattern for spinal motoneurons in the animals studied.

To analyze the response of IFNγ-KO neurons to axotomy, only those cells in lamina IX, sectioned at the nuclear plane, were counted and classified with regards to cellular morphology. Neurons darkly stained, which exhibited shrinkage of cytoplasm and signs of vacuolization, were classified as degenerating cells. The number and percentage of presumed motoneurons showing light microscopic features of degeneration in each section was obtained.

### Electron microscopy

The ventral horn area containing motoneurons in lamina IX was trimmed and ultrathin sections from L4-L6 segments were collected on formvar-coated copper grids, counterstained with uranyl acetate and lead citrate, and examinated in a Tecnai G2 Spirit Twin (FEI, Hillsboro, OR) transmission electron microscope operated at 80 kV. Neurons were photographed using an attached, side mounted, Gatan Orius SC1000B digital camera (Gatan, Inc, Pleasanton, CA) under different magnifications, and the digital images were used for ultrastructural analysis. Neurons with a large cell body (≥ 35 μm in diameter) found in the sciatic motoneuron pool and cut at the nuclear plane, were identified as alpha motoneurons by the presence of C-type boutons. For qualitative classification of bouton types, serial images of the entire soma membrane length were mounted sequentially using the Adobe Photoshop software. Boutons were classified into three different synaptic types [16]: F-type (with flattened synaptic vesicles), S-type (with spherical synaptic vesicles) and C-type (with subsynaptic cistern). For each neuron, we calculated the number of synaptic terminals per 100 μm of cell membrane and the percent of membrane length covered by terminals using the measurement tool of the Image Tool software. The distance between consecutive nerve terminals covering the neurons was also determined. A total of 40 alpha-motoneurons (2 neurons per animal in two groups of five animals: C57BL/6J unlesioned/lesioned side, IFNγ-KO unlesioned/lesioned side) were analyzed.

### Statistical analysis

All quantitative data are presented as mean ± SEM, and differences between groups were considered significant when the P-value was < 0.05(*). Statistical analysis was performed by using analysis of variance (ANOVA) with Holm-Sidak method (Sigmastat 3.1, Systat software, Inc., Point Richmond, CA) for parametric data or Mann-Whitney U test for non-parametric data.

## Results

In the present study, we analyzed the size distribution and morphological characteristics of neurons localized in lamina IX of the lumbar spinal cord from adult C57BL/6J (wild type) and IFNγ-KO (mutant) mice subjected to left sciatic nerve transection. Histochemical processing allowed for initial light microscopic analysis of sections depicting neurons within the ventrolateral horn. The light microscopy sections containing such presumed motoneurons were subsequently processed for ultrastructural analysis.

### Morphometric changes in the absence of IFNγ

**Figure 1:**
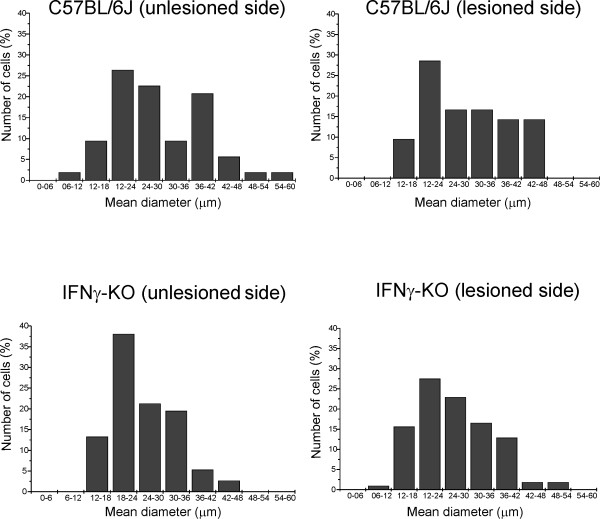
**Motoneuron somata mean diameter (μm), one week after the unilateral transection of the sciatic nerve in C57BL/6J and IFNγ-KO mice**. (A) Bimodal distribution in the C57BL/6J on the unlesioned side, with a clear through at 30-36 μm. (B) Lesioned side with non-bimodal size distribution. (C) Size distribution in the IFNγ-KO on the unlesioned side, the cell diameter is especially concentrated at 18-24 μm. (D) Lesioned side of the IFNγ-KO mice also showed a non-bimodal size distribution.

The quantitative data of the analyzed cells are shown in Figure 1. The mean diameter of neuronal somata ranged from 10.94 μm to 55.78 μm. The neurons demonstrated a bimodal size distribution in the wild type unlesioned side, with the mean cell body diameter exhibiting a trough at 30-35 μm and modes at 18 μm and 36 μm. On the lesioned side, the shape of the size distribution was altered and appeared as a non-bimodal distribution. Both the lesioned and unlesioned sides of the mutant mice showed the presence of only one peak with a mean soma diameter at 18 μm. The neuronal soma size in the mutant animals was smaller than corresponding measurements in the wild type. The statistical analysis thus showed that the differences in cell diameter values were significantly influenced by the gene mutation (p < 0.05), but it was not further altered by the peripheral lesion in mutant mice.

### Neuronal damage in IFNγ deficient mice

**Figure 2:**
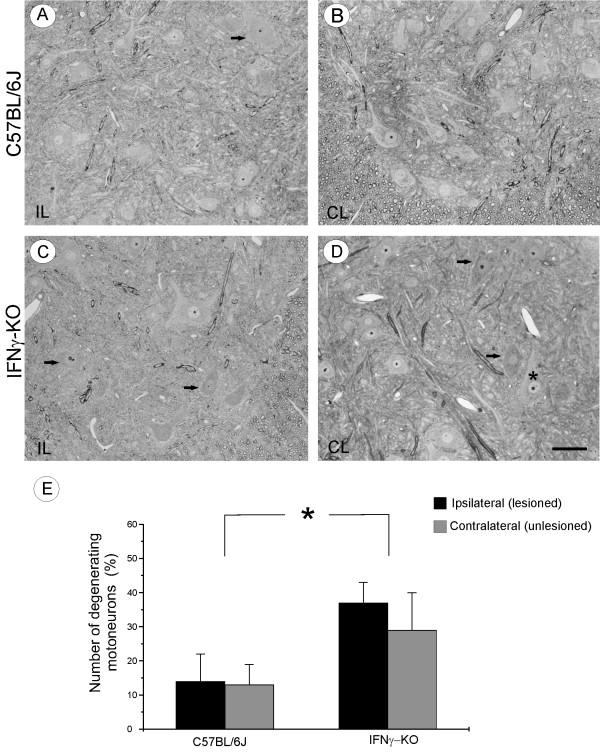
**Light photomicrographs of spinal cord ventral horn of C57BL/6J and IFNγ-KO mice from semithin sections stained with toluidine blue**. (A) Ipsilateral (lesioned) side and (B) contralateral (unlesioned) side of C57BL/6J mice. (C) IFNγ-KO ipsilateral side shows some motoneurons with hyperchromatic changes in the cytoplasm and nuclei (arrows). (D) IFNγ-KO contralateral side with pyknotic neurons found among at normal neurons (asterisk). (E) The number of degenerated motoneurons in axomized animals. Scale bar: 50 μm

In order to assess changes in cell morphology, semithin sections of wild type and mutant mice spinal cords were observed by light microscopy. In mutant mice a higher number of pyknotic neurons were found compared to corresponding counts in wild type animals (Figure 2A and 2B) and, in some cases, the nuclei also appeared darkly stained. Additionally, hyperchromatic changes in the cytoplasm and irregularly shaped cells were seen. Also, regarding size, a clear decrease due to shrinkage was encountered in the mutant series (Figure 2C). On the control side, it was also possible to identify some cells with pyknotic nuclei and cytoplasm intermingled with normal appearing neurons (Figure 2D).

Mutant animals showed a greater proportion of degenerating cells per section analyzed when compared to wild type mice (mutant lesioned side, 3.62 ± 0.99; mutant unlesioned side 2.43 ± 1.14; wild type lesioned side, 0.8 ± 0.69; wild type unlesioned side, 0.7 ± 0.42; p < 0.05). The values are expressed as the mean number of sampled degenerating neurons present in the motor nucleus in unlesioned mice. Additionally, the proportion of dying neurons in mutant mice was also statistically greater than in wild type mice. (ipsilateral IFNγ-KO, 37.64 ± 5.95%; contralateral IFNγ-KO, 29.28 ± 10.73%; ipsilateral C57BL/6J, 13.94 ± 8.55%; contralateral C57BL/6J, 13.00 ± 5.84%; p < 0.05) (Figure 2E).

### Neuronal degeneration in the absence of IFNγ

**Figure 3:**
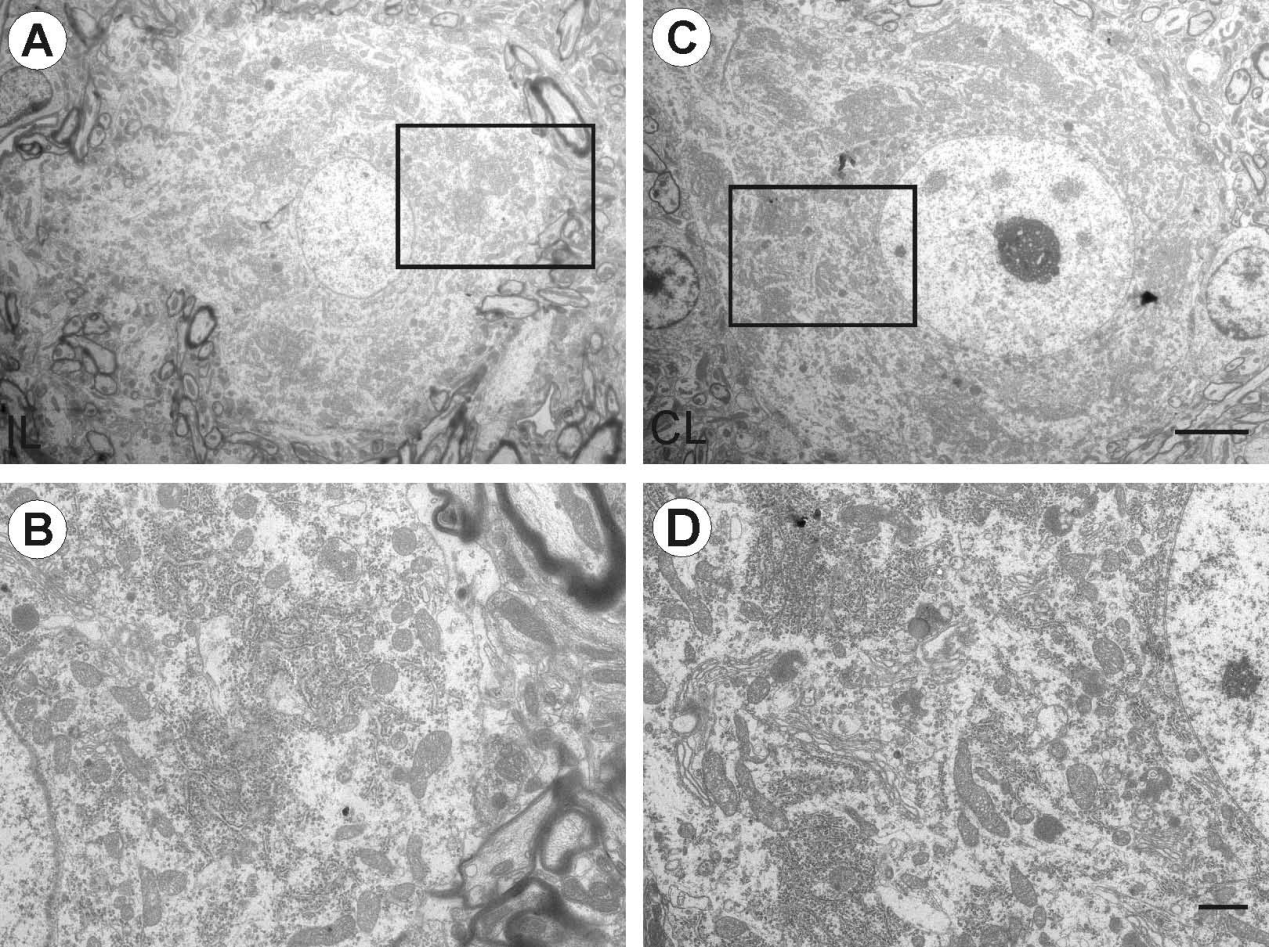
**Electron photomicrographs of spinal cord motoneurons of C57BL/6J mice**. (A) One α-motoneuron found on the ipsilateral side. The boxed area is shown at higher magnification in (B). Some synaptic terminals are retracted; the cytoplasm and nuclei have a normal appearance. (C) contralateral α-motoneuron. (D) Higher magnification shows cytoplasmatic organelles visible and well organized rough endoplasmic reticulum (RER), Golgi apparatus (GA) and mitochondria. Scale bar: 5 μm (A and C), 1 μm (B-D).

Following sciatic nerve axotomy in wild type mice, degenerating neurons were rarely found. In this sense, the majority of cells showed intact nuclear and cell membranes, a nucleus with evenly distributed chromatin, and no cytoplasmic alterations. The motoneurons from the lesioned side showed the classical changes associated with the axotomy. For instance, motoneurons showed signs of synaptic terminal retractions (Figure 3A), and somata presented features of chromatolysis. Sections from wild type operated side (Figure 3B) revealed several nerve terminals partially or totally displaced from the motoneuron cell body membrane. The ultrastructure also showed intact cytoplasmic organelles in presumed motoneurons on both the axotomized and unlesioned sides (Figure 3C). For instance, a well developed rough endoplasmic reticulum (RER) with rosettes of polyribosomes, Golgi apparatus (GA) containing parallel stacks of empty cisterns, and mitochondria exhibiting normal electron density and morphology were readily identified in the large ventral horn neurons of wild type mice (Figure 3D).

**Figure 4:**
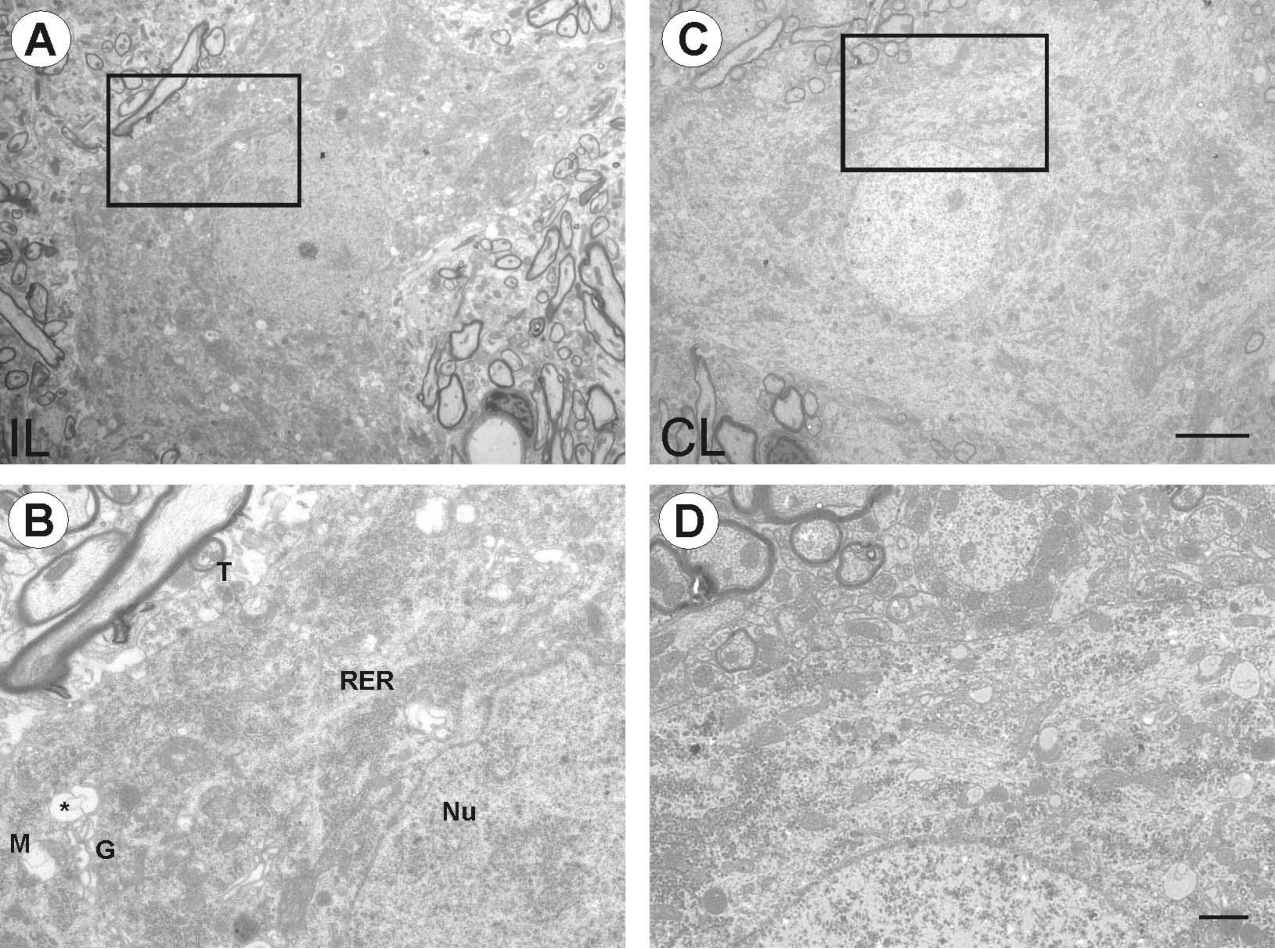
**Electron photomicrographs of spinal cord motoneurons of IFNγ-KO mice**. (A) α-motoneurons in the lesioned side. Boxed area is shown at higher magnification in (B). The cytoplasm and nuclei are more electron dense and cytoplasmatic organelles are disorganized. Note the presence of numerous vacuoles in the perikaryon (asterisk) and the irregular contour of nuclear membrane. (C) α-motoneurons in the contralateral side. (D) Higher magnification shows the cell less electron dense and with some vacuoles in the cytoplasm. T, synaptic terminal; M, mitochondria; RER, rough endoplasmic reticulum, Nu, nucleus; G, golgi apparatus. Scale bar: 5 μm (A and C), 1 μm (B-D).

In contrast, qualitative ultrastructural observations revealed in the mutant animals, even on the non-operated side, a higher number of neurons with severe morphological changes. Some of these alterations were also evident under light microscopy. Ventral horn neurons showed distinct morphology related to degeneration and cell death, including hyperchromatic changes in the nucleus, cytoplasm, and cytoplasmic organelles. Motoneurons on the lesioned side showed a decrease in their synaptic covering (Figure 4A). As a result of the modifications in the nucleus and cytoplasm, such cells became intensely electron dense and internally disorganized. Additionally, vacuoles of various sizes were found in the cytoplasm. In some neurons it was possible to see the nuclear chromatin aggregated into several clumps. Electron micrographs of higher magnification (Figure 4B) showed invaginations of nucleolemma indicating the shrinkage of the nucleus. Mitochondria were darkly stained and dilated, cisternae of rough endoplasmic reticulum were swollen and ribosomes appeared free in the cytoplasm.

**Figure 5:**
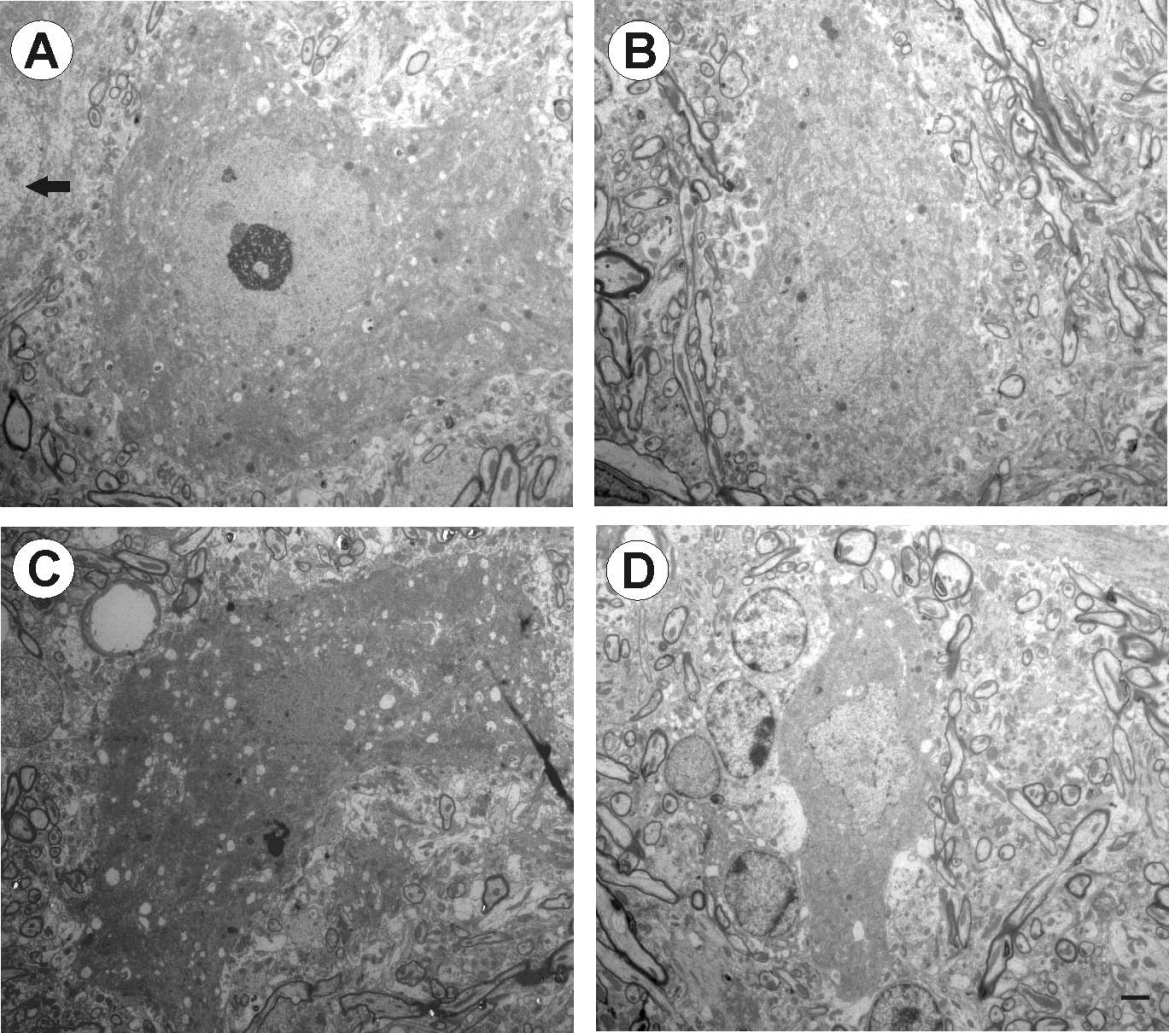
**Electron photomicrographs showing degenerated neurons in IFNγ-KO mice**. (A) One abnormal motoneuron amongst of normal cell (arrow). Cytoplasm and nuclei are electron dense; there are invaginations in the nuclear and cytoplasmic membranes and presence of many vacuoles in the cytoplasm. (B) The cell body appears shrunken and the nuclear envelope fragmented. (C) Darkly stained cytoplasm, condensed nuclear chromatin, and some organelles are hardly visible. (D) Glial cells are in close contact with a degenerating neuron. Note phagocytes of ingested material in the cytoplasm of microglial cells. Scale bar: 2 μm.

**Figure 6:**
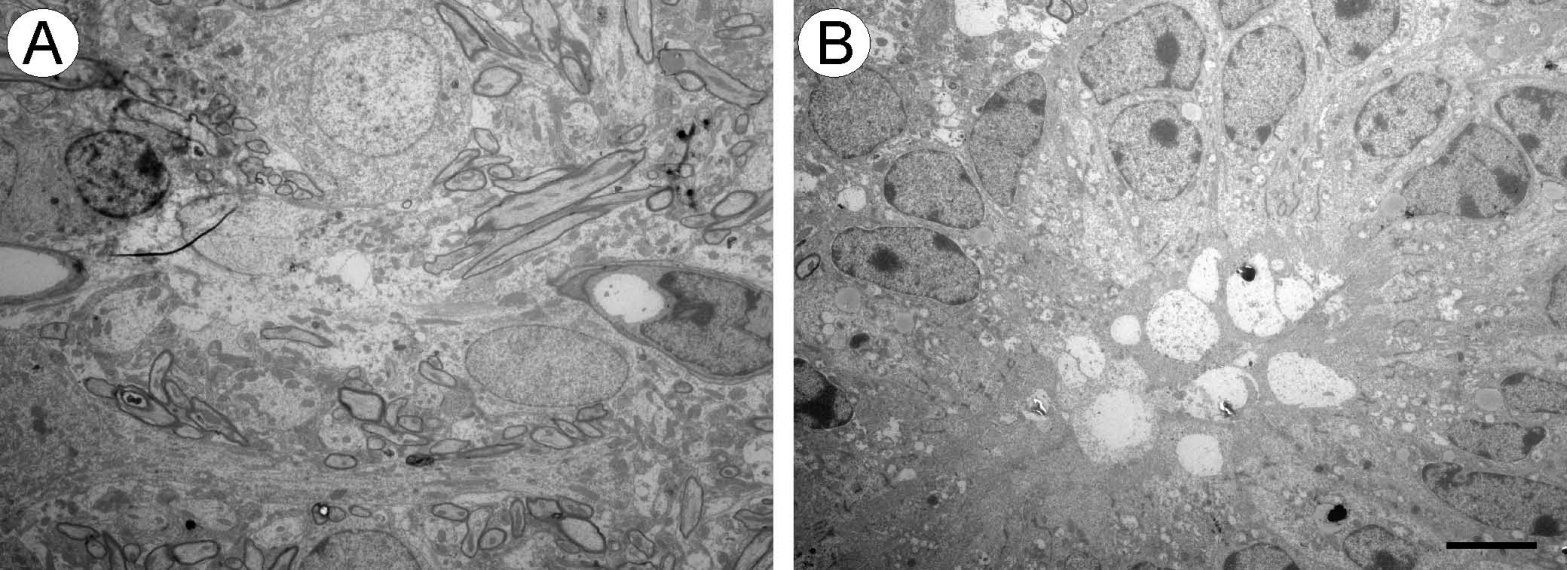
**Electron micrograph showing two representative dying neurons in layer IX of spinal cord ventral horn of IFNγ-KO mice**. (A) Dying neuron and the presence of glial cells in close contact with cell debris. (B) Many microglial cells ingesting a degenerating neuron. Scale bar: 5 μm.

Healthy motoneurons from the unlesioned side in mutant mice showed synaptic terminals in apposition to the post-synaptic membrane, but also some retractions (Figure 4C). Although the cells on this side appeared less electron dense and the nucleus was detectable, subcellular alterations were seen. Under high magnification, many vacuoles in the cytoplasm were encountered, and dilation of mitochondrial cristae were identified (Figure 4D). Abnormal neurons were found adjacent to normal appearing cells in the same tissue section (Figure 5A). Discontinuity of plasma membranes and nucleolemma was also observed in some neurons (Figure 5B). In cells with hyperchromatic cytoplasm and nucleus with condensed chromatin, the nuclear envelope became indistinguishable. In the cytoplasm, RER was very sparse, GA appeared dilated and many vacuoles were observed (Figure 5C). Some glial cells were visible close to the neuronal cell body, and microglial cells showed vacuoles containing ingested material in the cytoplasm (Figure 5D). Neuronal lysis with surrounding glial cells was also detected in the anterior horn of the spinal cord (Figure 6A and 6B) suggesting high phagocytic activity.

### Characterization of neuronal death in IFNγ deficient adult mice

**Figure 7:**
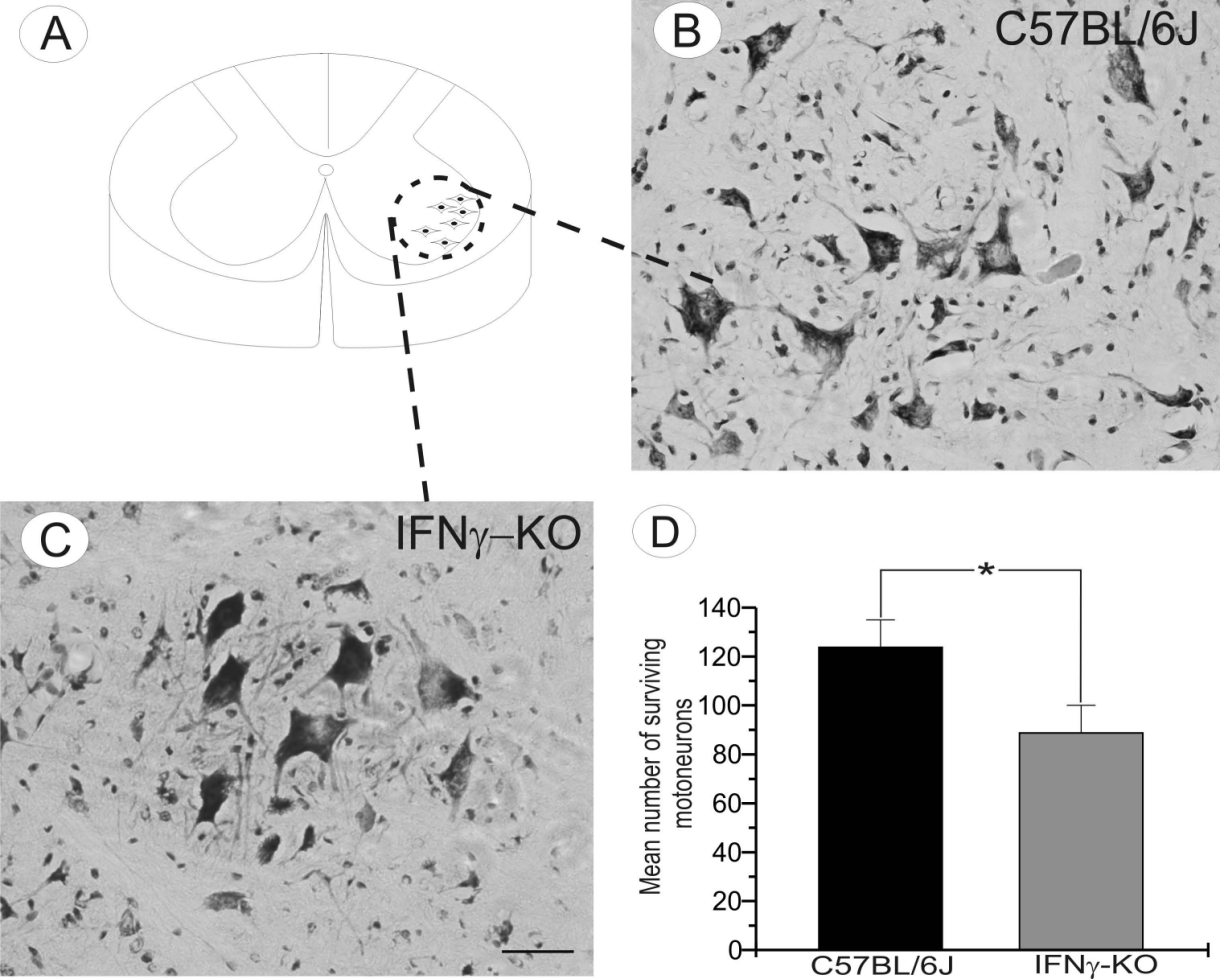
**Motoneuron degeneration in IFN-KO mice**. (A) The schematic drawing of spinal cord section at the lumbar level demonstrating the sciatic motoneurons pool. (B-C) Representative images showing motoneuron cell bodies in unlesioned C57BL/6J and IFNγ-KO animals. (D) Graphical representation of spinal motoneuron survival. Note a significant reduction in number of motoneurons in IFNγ-KO mice. Scale bar 50 μm

**Figure 8:**
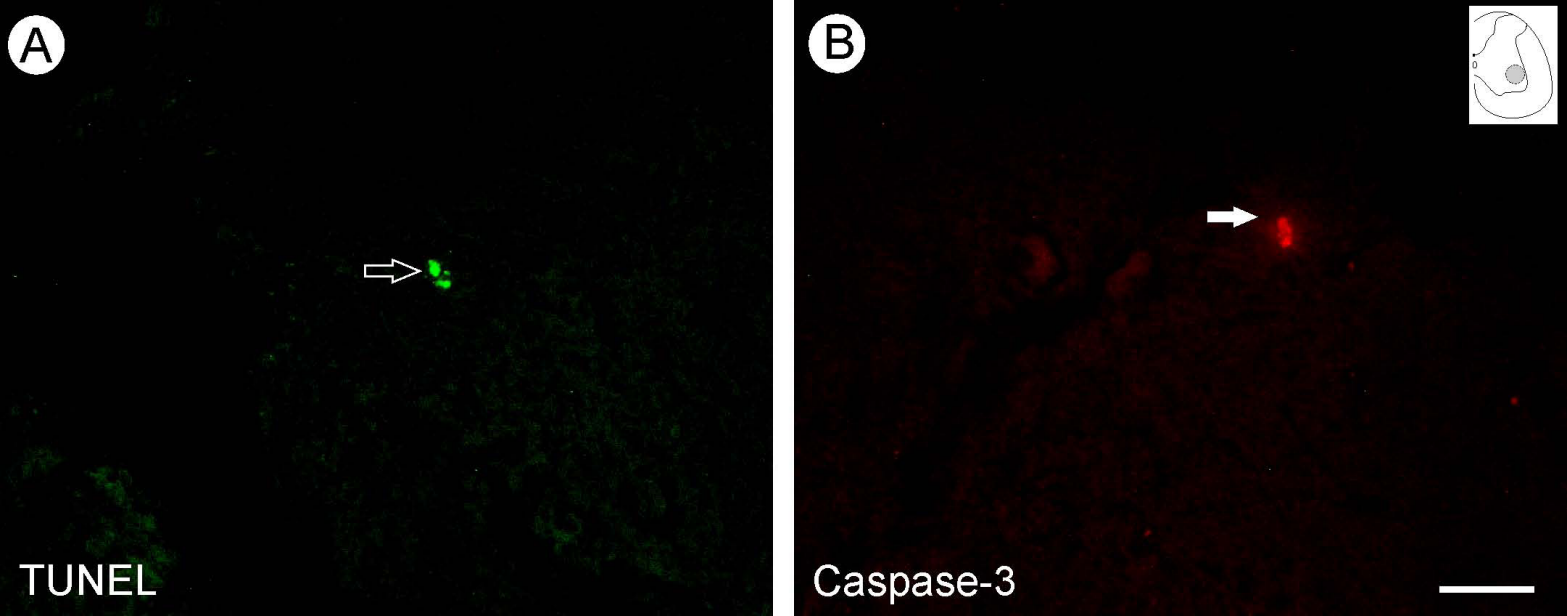
**Absence of IFNγ results in neuronal death**. (A) TUNEL labeled neuron (arrow) within the motor nucleus indicates the development of apoptosis. (B) Representative immunofluorescence image showing a caspase-3 positive motoneuron in an IFNγ-KO unlesioned mouse. Scale bar 50μm.

We have previously shown that IFNγ absence in adult mice subjected to axotomy resulted in several neuronal morphologic and morphometric changes. To evaluate whether the neuronal damage was mediated by the lack of IFNγ itself, the number of ventral horn neurons was quantified following Nissl-staining in unoperated animals. The mutant mice showed a lower number of surviving neurons in the lamina IX when compared to the wild type animals (89.00 ± 11.53 and 124.27 ± 11.15 per 100 alternate sections analyzed, respectively; p < 0.05, Figure 7). For identification of cell death, TUNEL and caspase-3 staining were performed in unoperated animals. Positive staining was found exclusively in mutant mice sections and is illustrated in Figure 8. The detection of caspase-3 immunolabeling in mutant mice reinforce the presence of apoptotic mechanisms involved in the motoneuron death described herein (Figure 8B). Therefore, we conclude that the reduction in the number of presumed motoneurons was attributable to the cellular degeneration and caused by the lack of IFNγ in those animals.

### Ultrastructural changes in the spinal cord of IFNγ-KO animals

**Figure 9:**
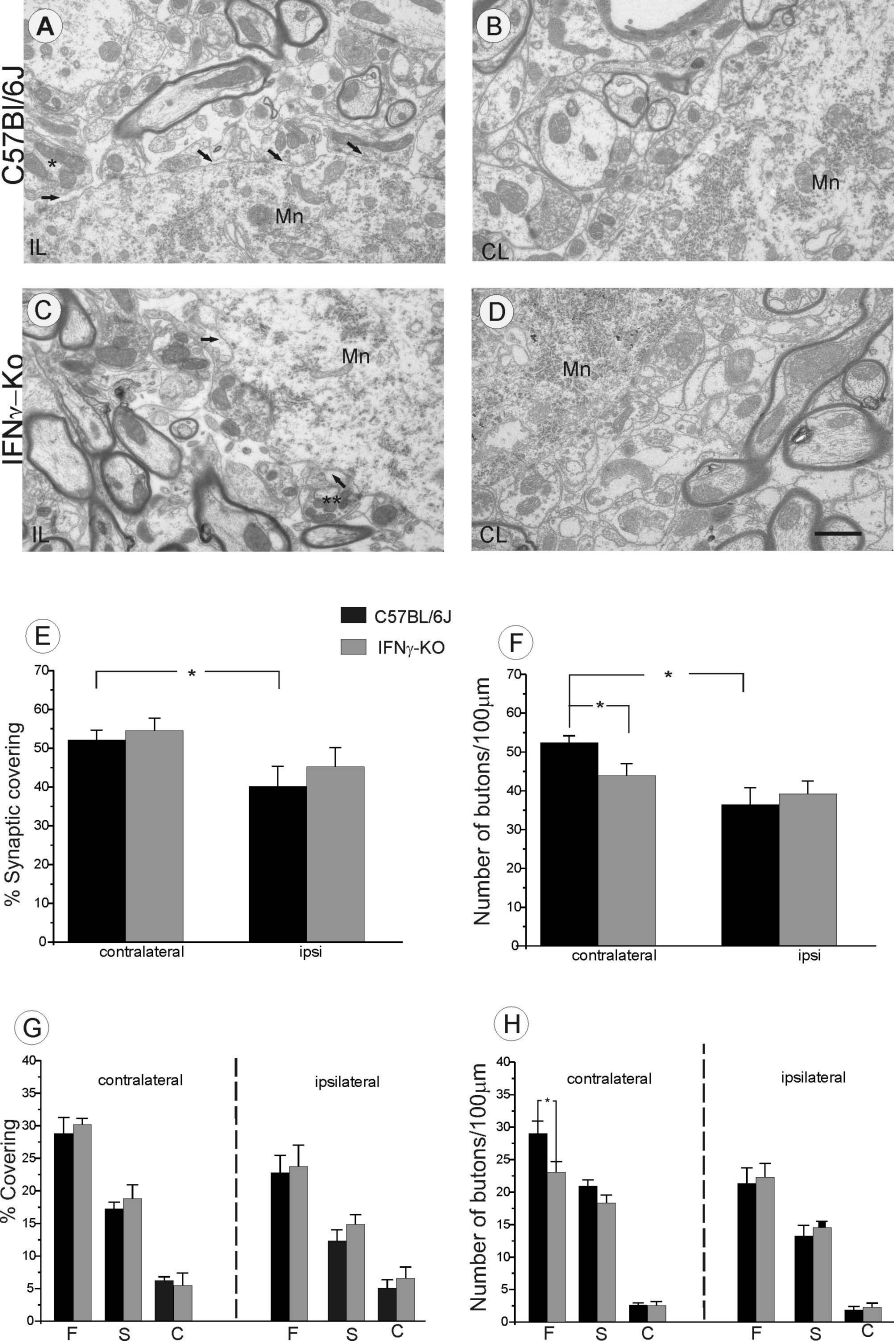
**Qualitative and quantitative ultrastructural analysis of every input to the surface of α-motoneurons one week after sciatic nerve axotomy**. (A) Synaptic retraction following axotomy in the C57BL/6J lesioned (ipsilateral) side. The arrows indicate the retraction of the nerve terminal from the surface of motoneurons. (B) Presynaptic terminals in apposition to motoneuron (Mn) membrane of C57BL/6J contralateral side. (C) Synaptic elimination following axotomy in IFNγ-KO lesioned side. *terminal totally retracted, ** terminal partially retracted. (D) Synaptic covering in the IFNγ-KO contralateral side. (E) Detailed analysis of the covering and number of synaptic boutons in IFNγ-KO mice. Those animals showed a lower detachment of synaptic terminals and a significant reduced number of synaptic terminals in apposition (F), regardless the nerve lesion. (G) Percentage of synaptic covering of F, S and C-terminals on unlesioned and lesioned sides. (H) Graphs of terminal numbers/100 μm of motoneurons membrane. Note a greater loss of F-type of terminals in IFNγ-KO mice. p < 0.05(*). Scale bar 1 μm.

Additional ultrastructural studies of the motoneurons revealed synaptic differences between the mutant and wild type strains (Figure 9). The quantitative analysis showed a reduced number of presynaptic terminals in mutant mice, regardless of the nerve lesion (unlesioned side 44.00 ± 3.04; lesioned side 39.23 ± 3.26/100 μm of membrane), compared with the unlesioned side of control animals (52.42 ± 1.76/100 μm of membrane, p < 0.05). The unlesioned side of the mutant mice presented a decreased number of terminals in apposition to the membrane of presumed motoneurons, which were not significantly further reduced following axotomy. Although mutant mice have shown a decreased number of terminals, no statistical differences could be observed among the studied strains regarding the synaptic input covering (p > 0.05).

An overall reduced number of presynaptic terminals in mutant mice may be attributed to a low number of F terminals (boutons containing flattened vesicles with glycine and/or GABA) in those animals. The mutant animals showed a significantly lower number of F-type boutons in apposition with ventral horn neurons on the unlesioned side compared to the corresponding neurons in the wild type mice (23.07 ± 1.61; 28.02 ± 1.68 respectively, p < 0.05). The number of F-type terminals on the lesioned side of the mutant mice did not change from the sciatic nerve lesion.

The analysis of the number of presynaptic S-type terminals (boutons containing spherical vesicles with glutamate) in apposition with ventral horn neurons showed no significant difference between the groups (mutant unlesioned side, 18.32 ± 1.21; wild type unlesioned side, 21.75 ± 0.89). The number of S-type boutons in apposition with presumed motoneurons on the lesioned side was similar in both samples (mutant, 14.55 ± 0.94; wild type, 13.27 ± 1.61).

**Figure 10:**
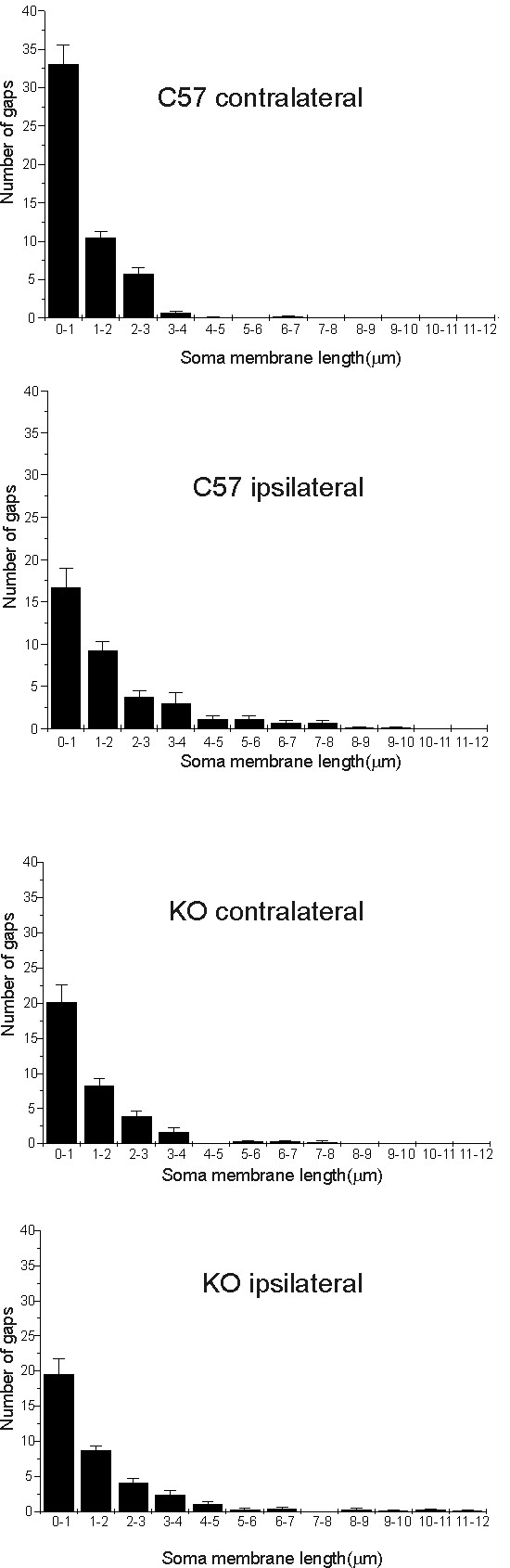
**Graphs showing the frequency distribution (in micrometers) of gaps between the terminals along the cell soma membrane of α-motoneurons**. (A-B) C57BL/6J animals showed a higher synaptic elimination one week after lesion. The gaps between clusters of boutons are possibly increased due to selective retractions of inputs. (C and D) IFNγ-KO mice showed a lower retraction of terminals after nerve lesion.

In order to analyze the pattern of terminal distribution along the motoneurons surface, the frequency of the gaps between clusters of boutons in apposition with the motoneurons somata was measured in each group (Figure 10). A lower number of gaps and a higher frequency of shorter intervals were observed between synaptic terminals in the mutant unlesioned side, than in the wild type mice (Figure 10C). Even with the sciatic nerve lesion (Figure 10D), the pattern of gap distribution was maintained and synaptic terminals remained clustered in apposition with the motoneuron somata.

## Discussion

The overlap between the immune and the nervous system pathways has become an intriguing subject over the last few years. The possibility that molecules originally seen as solely related to the immune response may have particular effects on synaptic plasticity and nervous system development has opened a new field of investigation. MHC I expression in the CNS development is an interesting example, since the same molecule that is used in the immune system to allow for intracellular infection surveillance is strongly related to the refinement of inputs in the visual system [17,18]. In this sense, IFNγ is the most potent inducer of major histocompatibility class I complex (MHC class I), which plays an important role in neuronal-glial communication, influencing the response to peripheral axotomy, directly affecting the synaptic plasticity process [19] and glial reactivity [20,21].

In the present study we have investigated the effects of the absence of IFNγ on the response to injury in mutant mice. Surprisingly, neuronal damage and cell shrinkage was depicted already in unlesioned mutant mice, and the axotomy itself did not worsen the degenerative process. These findings indicate that IFNγ expression is necessary to the survival of large cells present within the ventral horn of the spinal cord. Also, the observation that the synaptic circuits of sampled motoneurons were altered indicates that IFNγ is necessary for the proper development and maintenance of the synaptic homeostasis in the motor column. It is possible that IFNγ functions in a paracrine way, stimulating the MHC I expression by neurons [2,6,12], and thereby influence the continuous refinement and adjustments of synapses that take place in the spinal circuits throughout life.

The absence of IFNγ has been previously studied in the CNS after facial nerve transection. Under such circumstances, no significant changes could be depicted in the brain stem one week after lesion, both regarding glial reactions and MHC I expression [21]. However, synapse immuno-reactivity and ultrastructural analysis were not carried out at that time. In this sense, it is possible that more subtle modifications could have been overseen. Also, the spinal cord microenvironment may be considered as containing a more complex network of inputs to alpha motoneurons that are estimated to receive up to 100,000 synapses. The ability to respond to and integrate such a large number of pre-synaptic terminals may be a critical issue, possibly dependent on MHC I expression and regulation, that is linked to IFN expression.In the present work, a careful analysis of neuronal degeneration was carried out at light and ultrastructural levels. We show that presumed motoneurons in the lower lumbar ventral horn exhibited a smaller soma size in the mutant mice, regardless of nerve lesion. Also, the mutant mice demonstrated a greater proportion of degenerating neurons in the ventral horn as compared to the wild type. Motoneuron counts revealed a large number of degenerating cells in mutant mice in comparison to the wild type strain. Neuronal death was mostly due to the IFNγ gene knock out itself, since it could not be enhanced by peripheral axotomy, as seen after sciatic nerve transection. In addition, the mutant animals without lesion showed a reduced number surviving motoneurons compared with wild type. The presence of TUNEL positive cells in these animals confirm that apoptotic mechanisms are related to the observed motoneuron death. Nevertheless, necrotic death cannot be excluded based on the ultrastructural observations. The findings reported herein reveal the importance of IFNγ for neuronal survival throughout the normal adult life span.

The results described herein are in line with previous studies demonstrating that soluble mediators, such as certain cytokines, may have a protective role in the injured nervous system [4,9]. In this regard, recent studies have provided evidence for the protective function of IFNγ in neuronal cells after viral infections [14] and excitotoxicity [22]. In the adult brain of mice, the IFNγ was also associated with an improved spatial cognitive performance [23].

No statistical difference regarding the input covering could be observed between mutant and control mice. However, an interesting feature of other ultrastructural motoneuron changes in mutant mice is the possible occurrence of apoptosis and cell death, although a small subset of neurons appeared to die via necrosis. This is supported by TEM observations that include the classical features of apoptosis, such as cell bubbling, nuclear fragmentation and cell shrinkage.

Such morphological alterations were present in the spinal cord ventral horn, and the degenerating cells were surrounded by glial elements, mostly microglial cells. Since several pre-synaptic terminals could be seen retracted close to the degenerating elements, such cells most possibly were motoneurons. It is important to emphasize, however, that cells may show intermediate morphological forms which range from apoptosis to necrosis [24-27], so that both pathways may coexist after injury or during degeneration.

Interferon gamma expression within the nervous system is classically associated with an inflammatory response after injury [11]. In this way, it is normally involved as a part of the physiological response to tissue damage and trauma. Under these circumstances, various cytokines are produced and released by infiltrating immune cells. Activated T-cells may cross the blood-brain barrier and interact with resident cells of the central nervous system (CNS) [4,28] by synthetizing factors, such as IFNγ, and to induce glial cells to produce cell surface molecules and mediators required for crosstalk between immune and brain cells. The present study, however, indicates an additional role to IFNγ expression in the CNS. Specifically, IFNγ may participate in the homeostasis of the synaptic circuitry during the normal life span [29,30]. This is possibly linked with unveiled mechanisms involving immune molecules as important players for synaptic plasticity. The MHC I expression is a recent example of such overlap between CNS and immune system mechanisms [28]. Regulation of these molecules, including IFNγ, seems to affect the stability of the architecture of the nervous system [6,31]. The absence of these elements may in turn lead to neural degeneration and loss of function.

Further studies will be necessary to better understand the above mechanisms, and identify novel targets for new efficient strategies to treat lesions and degeneration of the nervous system.

## Conclusions

Altogether, the present results suggest that IFNγ may be neuroprotective and its absence results in neuronal death, which is mostly associated with morphological signs of apoptosis. Also, the lack of IFNγ alters the synaptic elimination process that follows a peripheral nerve lesion.

## List of abbreviations

CNS: central nervous system; GA: golgi apparatus; IFNγ-KO: interferon gamma knock out; MHC I: major histocompatibility complex of class; NK: natural killer; RER: rough endoplasmic reticulum; TEM: Transmission electron microscopy.

## Competing interests

The authors declare that they have no competing interests.

## Authors' contributions

ALRO and LAH provided the study concept, design and supervision. SCSV participated on the experimental design and acquisition of data. All authors provided analysis and interpretation and wrote the manuscript. All authors read and approved the final manuscript.
